# Predicting sales and cross-border e-commerce supply chain management using artificial neural networks and the Capuchin search algorithm

**DOI:** 10.1038/s41598-024-62368-6

**Published:** 2024-06-10

**Authors:** Lun Xie, Jiaquan Liu, Wei Wang

**Affiliations:** 1 School of Journalism and Communication, Shanghai University, Shanghai, 200072 China; 2 Anhui Business and Technology College, Wuhu, 231131 Anhui China

**Keywords:** Predicting sales, Cross-border e-commerce, Artificial neural networks, Capuchin search algorithm, Internet of Things, Computational science, Computer science, Information technology

## Abstract

E-commerce provides a large selection of goods for sale and purchase, which promotes regular transactions and commodity flows. Efficient distribution of goods and precise estimation of customer wants are essential for cost reduction. In order to improve supply chain efficiency in the context of cross-border e-commerce, this article combines machine learning approaches with the Internet of Things. The suggested approach consists of two main stages. Order prediction is done in the first step to determine how many orders each merchant is expected to get in the future. In the second phase, allocation operations are conducted and resources required for each retailer are supplied depending on their needs and inventory, taking into account each store’s inventory as well as the anticipated sales level. This suggested approach makes use of a weighted mixture of neural networks to anticipate sales orders. The Capuchin Search Algorithm (CapSA) is used in this weighted combination to concurrently enhance the learning and ensemble performance of models. This indicates that an effort is made to reduce the local error of the learning model at the model level via model weight adjustments and neural network configuration. To guarantee more accurate output from the ensemble model, the best weight for each individual component is found at the ensemble model level using the CapSA method. This method yields the ensemble model’s final output in the form of weighted averages by choosing suitable weight values. With a Root Mean Squared Error of 2.27, the suggested technique has successfully predicted sales based on the acquired findings, showing a minimum decrease of 2.4 in comparison to the comparing methodologies. Additionally, the suggested method’s strong performance is shown by the fact that it was able to minimize the Mean Absolute Percentage Error by 14.67 when compared to other comparison approaches.

## Introduction

As living standards rise and electronic networks become more and more ingrained in daily life, cross-border e-commerce is beginning to take center stage in the retail industry. The emergence of worldwide electronic commerce has accelerated the growth of the logistics industry. This has led to the emergence of a serious issue with the ongoing supply of cold chain goods in global electronic commerce^1,2^. Unbalanced order amounts, extensive pricing fluctuations, and extensive purchase and allocation are examples of potential causes of supply chain imbalances. If it occurred, the whole e-commerce system would be shut down. In order to guarantee that commodities can travel swiftly and dependably across an entire supply chain and can respond swiftly in the event that the chain is out of balance, it is imperative to study supply inventory management in cross-border electronic commerce networks^3^. The network of individuals, businesses, associations, resources, protocols, and technological advancements involved in creating and advertising a product is referred to as the “supply chain”.

The extraction of raw materials marks the beginning of the process, which concludes when the consumer gets the finished item^4^. According to supply chain cooperation^5^, in order to improve the logistical service experience of clients in the e-commerce industry chain, goods must be pre-stocked in regional warehouses of multiple worldwide marketplaces. In this method, the logistical time might be significantly decreased. However, since the manufacture and sales of e-commerce products are worldwide, cross-border e-commerce businesses need additional time to prepare for things like transportation, quality control inspections at customs, and commodity procurement. Big data analysis tools and methodologies are thus often used to forecast commodity sales for online commerce, providing the data basis for supply chain management and the technical foundation of global supply chains for cross-border e-commerce firms^6^. In addition to the volume and diversity of transaction data, a number of additional factors also affect sales estimates because of the complexity of the cross-border e-commerce sector^7–9^. Therefore, taking into consideration a variety of elements in sales forecasting remains a problem for e-commerce enterprises looking to increase forecasting accuracy and efficiency^6^.

Numerous new technologies have altered our life' course in the previous ten years. The 5G Internet of Things is a worldwide network built on a common communication protocol that aims to gather data from the real world and execute useful applications^10–12^. Using 5G IoT networks, the supply chain for international e-commerce is transformed into a value-added chain. Value is added to commodities in the supply chain throughout the processing, packing, shipping, and tracking phases. Related businesses in the finance, logistics, and technology industries also benefit. Manufacturers, distributors, retailers, suppliers, and customers make constitute a large e-commerce supply chain that should be managed by an intelligent IoT platform^13^.

This study suggests combining machine learning and the Internet of Things to improve supply chain performance in CBEC. The approach consists of two stages: order prediction and resource allocation. The method makes use of weighted neural networks and the CapSA to improve model performance both at the learning and ensemble levels. The ensemble model uses the CapSA approach to determine the optimal weights for each component, while the learning model utilizes model weight modifications and neural network topologies to decrease local error. The specifics of the novel features of the suggested approach are up for debate in this study. The first innovation consists of the weighted ensemble model that is proposed using the CapSA and Artificial Neural Networks (ANN). The neural networks in this model are dynamically structured, and the CapSA is used to dynamically modify their configuration. A reactive approach to enhancing supply chain performance is presented as the second innovation. This approach is based on data gathered using machine learning models. Utilizing real-time data via the Internet of Things framework to enable more efficient decision-making inside the supply chain system is the third innovation. Lastly, by reducing order fulfillment and product delivery times, the suggested model—which is predicated on these innovations—can raise customer satisfaction levels. The intended paper's main contributions are as follows:

- Introducing a new predictive model for electronic commerce supply chain management.
- Presenting a framework for integrating real-time IoT data into electronic commerce supply chain management.
- Proposing a proactive approach to inventory management based on predicted demand for each retailer in the electronic commerce system.
- Enhancing the order fulfillment process through the integration of real-time data and analysis of predictions made by machine learning models.

The paper is continued in this paragraph: Our analysis's second part was devoted to relevant studies. The supplies and procedures were provided in the third segment. The results have been assessed in the fourth part, and a conclusion has been drawn in the fifth.

## Related research

Related research is covered in this section from a variety of views. Li et al.^14^ studied the application of big data mining and analysis in cross-border e-commerce Resource Management System (ERP) systems to anticipate product sales and manage inventories. They developed a database of crucial characteristics using internet search engines and demonstrated that relevant indexes were more successful than other optimization approaches. A two-step clustering technique and an A-XGBoost model for linear and nonlinear forecasting were employed in Ji et al.’s^6^ suggested C-A-XGBoost forecasting model, which incorporates sales attributes and data series trends. Using IoT monitoring techniques and multiobjective decision-making, Xia et al.^13^ presented an ideal management and coordination approach for maximizing the performance of a cross-border e-commerce supply chain. The recommended approach showed excellent internal consistency, with a Cronbach's alpha of above 80%. Sales, warehousing, and R&D localization are the three categories of supply chain localizations that Wang et al.^15^ distinguished for export markets via cross-border e-commerce. According to the report, understanding supply chain financing, bonded warehouses, and foreign warehouses is essential for developing new business models. An intelligent agent framework was created by Shen et al.^16^ to choose providers in a multi-product context that offer combinatorial advantages.

Jiang et al.^17^ examined project-driven supply networks in the setting of asymmetric information and decentralized decision-making using an agent-based approach and evolutionary computing. In order to examine how government regulations and individual sustainable product purchases affect supplier greenness choices and marketplace growth elements, Li et al.^18^ developed a three-way agent framework. For cross-border e-business import supply chains, Li et al.^19^ developed a deep learning-based price choice algorithm that combines rational expectation equilibrium analysis and outperforms traditional decision tree algorithms in terms of accuracy and recall.

According to Qi et al.^20^, the Central Bank of East Asia (CBEC) entrance method was selected because it needed less in the form of asset investment, labor needs, logistics, and storage expenses. It also decreased uncertainty and enhanced trust. The machine learning-based framework for intelligent Cross-Border E-Commerce (CBEC) developed by Wang et al.^21^ employs an ensemble system and particle swarm optimization. This approach results in enhanced efficiency and a reduced average absolute error of 2.54. Using a game-theoretical model, Zhang et al.^22^ looked into the optimal information-sharing strategy in a cross-border e-commerce supply chain with variable tax rates. High-quality elasticity promoted voluntary sharing, which lessened the effect of tax increases on incomes, according to the research.

By adding product assortment hierarchy data, training an LSTM, and proposing a pre-processing framework, Bandara et al.^23^ improved E-commerce sales estimates, beating previous methods on category and super-departmental datasets. A machine learning prediction technique was proposed by Leung et al.^24^ that used historical retailer data and integrated it with optimization or heuristic methods to estimate near-realtime order delivery in e-commerce distribution facilities. A neural network model for forecasting e-commerce demand was introduced by Cai et al.^25^ using multimodal data and spatial feature fusion. Order sequence, customer emotional, and face value aspects were all successfully retrieved by the model. Singh et al.’s study^26^ focused on using machine learning to predict e-commerce sales, highlighting four widely used algorithms in particular. A web application was developed using the best model with a comparable prediction range, guaranteeing precise and trustworthy sales forecasting for e-commerce platforms. Table 1 summarizes the literature review.

**Table 1:** Summary of the literature review.

Ref	Year	Objective	Method	Limitation
Li et al.^14^	2019	Predict product sales and optimize inventory	Big data mining and analysis for sales prediction	Relies on internet search data, may not capture all relevant factors
Ji et al.^6^	2019	Sales forecasting for cross-border e-commerce	C-A-XGBoost model with two-step clustering	Limited to sales features and data series trends, may not capture external factors
Xia et al.^13^	2021	Improve CBEC supply chain performance	IoT tracking and multi-objective decision-making	Focuses on internal consistency, may not address overall efficiency
Wang et al.^15^	2018	Analyze supply chain localization in CBEC	Identify key capabilities for business model innovation	Lacks quantitative analysis of performance improvement
Shen et al.^16^	2022	Select suppliers in multi-product setting	Intelligent agent framework	Not specific to CBEC, may not consider cross-border challenges
Jiang et al.^17^	2021	Investigate project-driven supply networks	Agent-based method and evolutionary computing	Complex model, may be computationally expensive for real-time applications
Li et al.^18^	2023	Analyze impact of green purchasing on suppliers	Three-way agent framework	Focuses on sustainability, may not directly address sales prediction
Li et al.^19^	2023	Design pricing decision algorithm for CBEC	Deep learning-based approach	Lacks comparison with other forecasting methods in CBEC context
Qi et al.^20^	2020	Explain rationale for selecting CBEC entry mode	Transaction cost theory	Doesn't address sales prediction or supply chain optimization
Wang et al.^21^	2023	Develop machine learning framework for CBEC	Ensemble system and particle swarm optimization	Achieves good accuracy but details of model not provided
Zhang et al.^22^	2022	Investigate information sharing in CBEC	Game-theoretical model	Focuses on information sharing strategy, not directly related to sales prediction
Bandara et al.^23^	2019	Enhance e-commerce sales forecasts	LSTM with product assortment data	Limited to forecasting within specific product categories
Leung et al.^24^	2020	Forecast near-real-time order arrivals	Machine learning for order arrival prediction	Doesn't address sales prediction over longer timeframes
Cai et al.^25^	2021	Predict e-commerce demand	Neural network model with spatial feature fusion and multimodal data	Model complexity may limit interpretability and scalability
Singh et al.^26^	2020	Utilize machine learning for e-commerce sales forecasting	Comparison of four common machine learning algorithms	Limited to comparing existing algorithms, doesn't propose a new approach

## Research methodology

This section presents a model for supply chain management in CBEC using artificial intelligence (AI). The approach provides resource provisioning by using a collection of ANNs to forecast future events. Prior to going into depth about this method, the dataset specifications utilized in this study are given.

### Data

The performance of seven active sellers in the sphere of international products trade over the course of a month was examined in order to get the data for this study. At the global level, all of these variables are involved in the bulk physical product exchange market. This implies that all goods bought by clients have to be sent by land, air, or sea transportation. In order to trade their items, each seller in this industry utilizes a minimum of four online sales platforms. Each of the 945 documents that make up the datasets that were assembled for each vendor includes data on the number of orders that consumers have made with that particular vendor. Each record's bulk product transactions have minimum and maximum amounts of 3, and 29 units, respectively. Every record is defined using a total of twenty-three distinct attributes. Some of the attributes that are included are order registration time, date, month, method (platform type used), order volume, destination, product type, shipping method, active inventory level, product shipping delay history indicated by active in the previous seven transactions, and product order volume history throughout the previous seven days. For each of these two qualities, a single numerical vector is used.

### Proposed framework

This section describes a CBEC system that incorporates a tangible product supply chain under the management of numerous retailers and platforms. The primary objective of this study is to enhance the supply chain performance in CBEC through the implementation of machine learning (ML) and Internet of Things (IoT) architectures. This framework comprises four primary components:

- *Retailers* They are responsible for marketing and selling products.
- *Common sales platform* Provides a platform for introducing and selling products by retailers.
- *Product warehouse* It is the place where each retailer stores their products.
- *Supply center* It is responsible for instantly providing the resources needed by retailers. The CBEC system model comprises N autonomous retailers, all of which are authorized to engage in marketing and distribution of one or more products. Each retailer maintains a minimum of one warehouse for product storage. Additionally, retailers may utilize multiple online sales platforms to market and sell their products.

Consumers place orders via these electronic commerce platforms in order to acquire the products they prefer. Through the platform, the registered orders are transmitted to the product's proprietor. The retailer generates and transmits the sales form to the data center situated within the supply center as soon as it receives the order. The supply center is responsible for delivering the essential resources to each retailer in a timely manner. In traditional applications of the CBEC system, the supply center provides resources in a reactive capacity. This approach contributes to an extended order processing time, which ultimately erodes customer confidence and may result in the dissolution of the relationship. Proactive implementation of this procedure is incorporated into the proposed framework. Machine learning methods are applied to predict the number of orders that will be submitted by each agent at future time intervals. Following this, the allocation of resources in the storage facilities of each agent is ascertained by the results of these forecasts. In accordance with the proposed framework, the agent's warehouse inventory is modified in the data center after the sales form is transmitted to the data center. Additionally, a model based on ensemble learning is employed to forecast the quantity of upcoming orders for the product held by the retailer. The supply center subsequently acquires the required resources for the retailer in light of the forecast's outcome. The likelihood of inventory depletion and the time required to process orders are both substantially reduced through the implementation of this procedure.

As mentioned earlier, the efficacy of the supply chain is enhanced by this framework via the integration of IoT architecture. For this purpose, RFID technology is implemented in supply management. Every individual product included in the proposed framework is assigned a unique RFID identification tag. The integration of passive identifiers into the proposed model results in a reduction of the system's ultimate implementation cost. The electronic device serves as an automated data carrier for the RFID-based asset management system in the proposed paradigm. The architecture of this system integrates passive RFID devices that function within the UFH band. In addition, tag reader gateways are installed in the product warehouses of each retailer to facilitate the monitoring of merchandise entering and departing the premises. The proposed model commences the product entry and exit procedure through the utilization of the tag reader to extract the distinct identifier data contained within the RFID tags. The aforementioned identifier is subsequently transmitted to the controller in which the reader node is connected. A query containing the product's unique identifier is transmitted by the controller node to the data center with the purpose of acquiring product information, including entry/exit authorization. Upon authorization of this procedure, the controller node proceeds to transmit a storage command to the data center with the purpose of registering the product transfer information. This registration subsequently modifies the inventory of the retailer's product warehouse. Therefore, the overall performance of the proposed system can be categorized into the subsequent two overarching phases:

1. Predicting the number of future orders of each retailer in future time intervals using ML techniques.
2. Assigning resources to the warehouses of specific agents based on the outcomes of predictions and verifying the currency of the data center inventory for each agent's warehouse. The following sub-sections will be dedicated to delivering clarifications for each of the aforementioned phases.

#### ML-based order volume prediction and optimization

The imminent order volume for each vendor is forecasted within this framework through the utilization of a weighted ensemble model. A direct proportionality exists between the quantity of prediction models and the number of retailers that participate in the CBEC system. In order to predict the future volume of customer orders for the affiliated retailer, each ensemble model compiles the forecasts produced by its internal learning models. The supplier furnishes the requisite supplies to each agent in adherence to these projections. Through proactive measures to alleviate the delay that arises from the reactive supply of requested products, this methodology maximizes the overall duration of the supply chain product delivery process. Utilizing a combination of FSFS and ANOVA, the initial step in forecasting sales volume is to identify which attributes have the greatest bearing on the sales volume of particular merchants. Sales projections are generated through the utilization of a weighted ensemble model that combines sales volume with the most pertinent features. The proposed weighted ensemble model for forecasting the order volume of a specific retailer trained each of the three ANN models comprising the ensemble using the order patterns of the input from that retailer. While ensemble learning can enhance the accuracy of predictions produced by learning systems, there are two additional factors that should be considered in order to optimize its performance even further.

- *Acceptable performance of each learning model* Every learning component in an ensemble system has to perform satisfactorily in order to lower the total prediction error by combining their outputs. This calls for the deployment of well-configured learning models, such that every model continues to operate as intended even while handling a variety of data patterns.
- *Output weighting* In the majority of ensemble system application scenarios, the efficacy of the learning components comprising the system differs. To clarify, while certain learning models exhibit a reduced error rate in forecasting the objective variable, others display a higher error rate. Consequently, in contrast to the methodology employed in traditional ensemble systems, it is not possible to designate an identical value to the output value of every predictive component. In order to address this issue, one may implement a weighting strategy on the outputs of each learning component, thereby generating a weighted ensemble system.

**Figure 1:**
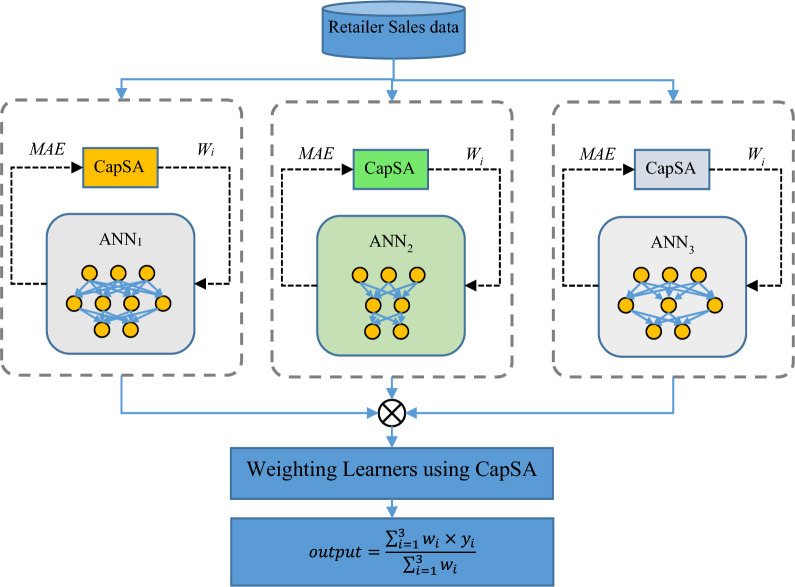
Operation of the proposed weighted ensemble model for predicting order volume.

CapSA is utilized in the proposed method to address these two concerns. The operation of the proposed weighted ensemble model for forecasting customer order volumes is illustrated in Fig. 1.

As illustrated in Fig. 1, the ensemble model under consideration comprises three predictive components that collaborate to forecast the order volume of a retailer, drawing inspiration from the structure of the ANN. Every individual learning model undergoes training using a distinct subset of sales history data associated with its respective retailer. The proposed method utilizes CapSA to execute the tasks of determining the optimal configuration and modifying the weight vector of each ANN model. It is important to acknowledge that the configuration of every ANN model is distinct from that of the other two models. By employing parallel processing techniques, the configuration and training of each model can be expedited. Every ANN model strives to determine the parameter values in a way that minimizes the mean absolute error criterion during the configuration phase. An optimal configuration set of learning models can be obtained through the utilization of this mechanism, thereby guaranteeing that every component functions at its designated level. After the configuration of each ANN component is complete, the procedure to determine the weight of the output of the predictive component is carried out. In order to accomplish this goal, CapSA is employed. During this phase, CapSA attempts to ascertain the output value of each learning model in relation to its performance.

After employing CapSA to optimize the weight values, the assembled and weighted models can be utilized to predict the volume of orders for novel samples. To achieve this, during the testing phase, input features are provided to each of the predictive components ANN1, ANN2, and ANN3. The final output of the proposed model is computed by averaging the weighted averages of the outputs from these components.

##### Feature selection

It is possible for the set of characteristics characterizing the sales pattern to contain unrelated characteristics. Hence, the proposed approach employs one-way ANOVA analysis to determine the significance of the input feature set and identify characteristics that are associated with the sales pattern. The F-score values of the features are computed in this manner utilizing the ANOVA test. Generally speaking, characteristics that possess greater F values hold greater significance during the prediction stage and are thus more conspicuous. Following the ranking of the features, the FSFS method is utilized to select the desired features. The primary function of FSFS is to determine the most visible and appropriate subset of ranked features. The algorithm generates the optimal subset of features by iteratively selecting features from the input set in accordance with their ranking. As each new feature is incorporated into the feature subset at each stage, the learning model's prediction error is assessed. The feature addition procedure concludes when the performance of the classification model is negatively impacted by the addition of a new feature. In such cases, the optimal subset is determined as the feature subset with the smallest error. Utilizing the resultant feature set, the ensemble system's components are trained in order to forecast sales volume.

##### Configuration of each ANN model based on CapSA in the proposed ensemble system

CapSA is tasked with the responsibility of identifying the most appropriate neural network topologies and optimal weight values within the proposed method. As previously stated, the ensemble model under consideration comprises three ANNs, with each one tasked with forecasting the forthcoming sales volume for a specific retailer. Using CapSA, the configuration and training processes for each of these ANN models are conducted independently. This section provides an explanation of the procedure involved in determining the optimal configuration and modifying the weight vector for each ANN model. Hence, the subsequent section outlines the steps required to solve the aforementioned optimization problem using CapSA, after which the structure of the solution vector and the objective function are defined. The suggested method's optimization algorithm makes use of the solution vector to determine the topology, network biases, and weights of neuronal connections. As a result, every solution vector in the optimization process consists of two linked parts. The first part of the solution vector specifies the network topology. Next, in the second part, the weights of the neurons and biases (which match the topology given in the first part of the solution vector) are determined. As a result, the defined topology of the neural network determines the variable length of the solution vectors in CapSA. Because a neural network might have an endless number of topological states, it is necessary to include certain restrictions in the solution vector that relate to the topology of the network. The first part of the solution vector is constrained by the following in order to narrow down the search space:

1. The precise count of hidden layers in any neural network is one. As such, the first element of the solution vector consists of one element, and the value of that element represents the number of neurons assigned to the hidden layer of the neural network.
2. The hidden layer of the neural network has a minimum of 4 and a maximum of 15 neurons.

The number of input features and target classes, respectively, determine the dimensions of the input and output layers of the neural network. As a result, the initial segment of the solution vector, known as the topology determination, solely specifies the quantity of neurons to be contained in the hidden layers. Given that the length of the second part of the solution vector is determined by the topology in the first part, the length of the first part determines the number of neurons in the neural network. For a neural network with *I* input neurons, *H* hidden neurons, and *P* output neurons, the length of the second part of the solution vector in CapSA is equal to $H\times (I+1)+P\times (H+1)$.

In CapSA, the identification of optimal solutions involves the application of a fitness function to each one. To achieve this goal, following the solution vector-driven configuration of the neural network's weights and topology, the network produces outputs for the training samples. These outputs are then compared to the actual target values. Following this, the mean absolute error criterion is applied to assess the neural network's performance and the generated solution's optimality. CapSA’s fitness function is thus characterized as follows:

1 $$MAE=\sum_{i=1}^{N}\left|{T}_{i}-{Z}_{i}\right|$$

In this context, N denotes the quantity of training samples, while Ti signifies the desired value to be achieved for the i-th training sample. Furthermore, the output generated by the neural network for the i-th training sample is denoted as Zi. The proposed method utilizes CapSA to ascertain a neural network structure capable of minimizing Eq. (1). In CapSA, both the initial population and the search bounds for the second portion of the solution vector are established at random [− 1, + 1]. Thus, all weight values assigned to the connections between neurons and biases of the neural network fall within this specified range. CapSA determines the optimal solution through the following procedures:

*Step 1* The initial population of Capuchin agents is randomly valued.

*Step 2* The fitness of each solution vector (Capuchin) is calculated based on Eq. (1).

*Step 3* The initial speed of each Capuchin agent is set.

*Step 4* Half of the Capuchin population is randomly selected as leaders and the rest are designated as follower Capuchins.

*Step 5* If the number of algorithm iterations has reached the maximum *G*, go to step 13, otherwise, repeat the following steps:

*Step 6* The CapSA lifespan parameter is calculated as follows^27^:

2 $$\tau ={\beta }_{0}{e}^{{\left(-\frac{{\beta }_{1}g}{G}\right)}^{{\beta }_{2}}}$$

where *g* represents the current number of iterations, and the parameters ${\beta }_{0}$, ${\beta }_{1}$, and ${\beta }_{2}$ have values of 2, 21, and 2, respectively.

*Step 7* Repeat the following steps for each Capuchin agent (leader and follower) like *i*:

*Step 8* If *i* is a Capuchin leader; update its speed based on Eq. (3)@@^27^:

3 $${v}_{j}^{i}=\rho {v}_{j}^{i}+\tau {a}_{1}\left({x}_{bes{t}_{j}}^{i}-{x}_{j}^{i}\right){r}_{1}+\tau {a}_{2}\left(F-{x}_{j}^{i}\right){r}_{2}$$

where the index j represents the dimensions of the problem and ${v}_{j}^{i}$ represents the speed of Capuchin i in dimension j. ${x}_{j}^{i}$ indicates the position of Capuchin i for the j-th variable and ${x}_{bes{t}_{j}}^{i}$ also describes the best position of Capuchin i for the j-th variable so far. Also, ${r}_{1}$ and ${r}_{2}$ are two random numbers in the range [0,1]. Finally, $\rho$ is the parameter affecting the previous speed, which is set to 0.7.

*Step 9* Update the new position of the leader Capuchins based on their speed and movement pattern.

*Step 10* Update the new position of the follower Capuchins based on their speed and the leader’s position.

*Step 11* Calculate the fitness of the population members based on Eq. (1).

*Step 12* If the entire population’s position has been updated, go to Step 5, otherwise, repeat the algorithm from Step 7.

*Step 13* Return the solution with the least fitness as the optimal configuration of the ANN model.

Once each predictive component has been configured and trained, CapSA is utilized once more to assign the most advantageous weights to each of these components. Determining the significance coefficient of the output produced by each of the predictive components ANN1, ANN2, and ANN3 with respect to the final output of the proposed ensemble system is the objective of optimal weight allocation. Therefore, the optimization variables for the three estimation components comprising the proposed ensemble model correspond to the set of optimal coefficients in this specific implementation of CapSA. Therefore, the length of each Capuchin in CapSA is fixed at three in order to determine the ensemble model output, and the weight coefficients are assigned to the outputs of ANN1, ANN2, and ANN3, correspondingly. Each optimization variable's search range is a real number between 0 and 1. After providing an overview of the computational methods employed in CapSA in the preceding section, the sole remaining point in this section is an explanation of the incorporated fitness function. The following describes the fitness function utilized by CapSA to assign weights to the learning components according to the mean absolute error criterion:

4 $$fitness=\frac{1}{n} \sum_{i=1}^{n}{T}_{i}-\frac{\sum_{j=1}^{3}{w}_{j}\times {y}_{j}^{i}}{\sum_{j=1}^{3}{w}_{j}}$$

where ${T}_{i}$ represents the actual value of the target variable for the *i*-th sample. Also, ${y}_{j}^{i}$ represents the output estimated by the ANN_j_ model for the *i*-th training sample, and w_j_ indicates the weight value assigned to the ANN_j_ model via the solution vector. At last, n describes the number of training samples.

A weight coefficient is allocated to each algorithm within the interval [0,1], delineating the manner in which that algorithm contributes to the final output of the ensemble model. It is crucial to note that the weighting phase of the learning components is executed only once, after the training and configuration processes have been completed. Once the optimal weight values for each learning component have been determined by CapSA, the predicted volume of forthcoming orders is executed using the trained models and the specified weight values. Once the predictive output of all three implemented ANN models has been obtained, the number of forthcoming orders is computed as follows by the proposed weighted ensemble model:

5 $$output=\frac{\sum_{i=1}^{3}{w}_{i}\times {y}_{i}}{\sum_{i=1}^{3}{w}_{i}}$$

Within this framework, the weight value (wi) and predicted value (yi) denote the ANNi model's assigned weight and predicted value, respectively, for the provided input sample. Ultimately, the retailer satisfies its future obligations in accordance with the value prediction produced by this ensemble model.

#### Supplying the resources of each agent’s warehouses based on prediction results

By predicting the sales volume of the product for specific retailers, it becomes possible to procure the requisite resources for each retailer in alignment with the projected sales volume. By ensuring that the supplier's limited resources are distributed equitably, this mechanism attempts to maximize the effectiveness of the sales system. In the following analysis, the sales volume predicted by the model for each retailer designated as i is represented by pi, whereas the agent's current inventory is denoted by vi. Furthermore, the total distribution capacity of the supplier is represented as L. In such a case, the supplier shall allocate the requisite resources to the retailer as follows:

1. *Sales volume prediction* Applying the model described in the previous part, the upcoming sales volume for each agent in the future time interval (*p*_*i*_) is predicted.
2. *Receiving warehouse inventory* The current inventory of every agent (*vi*) is received through supply chain management systems.
3. *Calculating the required resources* The amount of resources required for the warehouse of each retailer is calculated as follows:
  6 $$S_{i} = \max \left( {0,p_{i} - v_{i} } \right)$$
4. *Calculating each agent’s share of allocatable resources* The share of each retailer from the allocatable resources is calculated by Eq. (7), (*N* represents the number of retailers):
  7 $${R}_{i}=\frac{{S}_{i}}{\sum_{j=1}^{N}{S}_{j}}$$
5. *Resource allocation* The supply center sends the needed resources for each agent according to the allocated share (*Ri*) to that agent’s warehouse.
6. *Inventory update* The inventory of every agent is updated with the receipt of new resources.

## Reteach finding

The software MATLAB 2021 was employed to execute the model that was proposed in this paper. In this investigation, a tenfold cross-validation strategy was implemented. Two phases were devoted to the evaluation of the proposed method. Field evaluation of the efficacy of a proposed method for an online sales system comprised the initial phase. The effectiveness of this proposed method in forecasting sales levels was then evaluated and contrasted to the approaches taken by Leung et al.^24^, Cai et al.^25^, and Singh et al.^26^ in the second phase.

### First phase: examination of the time required to fulfill orders

During this stage, the efficacy of the suggested approach was assessed by utilizing data obtained from legitimate retailers. In the beginning, an assessment was made of the six retailers' performance in the dataset regarding order completion time and delivery time on an hourly basis over a period of time (supposed to be one month) without employing the suggested methodology. Using the proposed framework, an analysis of the order completion time for the same retailers during the same one-month period was subsequently conducted. The results of these evaluations were overtly disclosed during phase two. By contrasting these two phases, the efficacy of the proposed method in accelerating or delaying order completion was determined.

**Figure 2:**
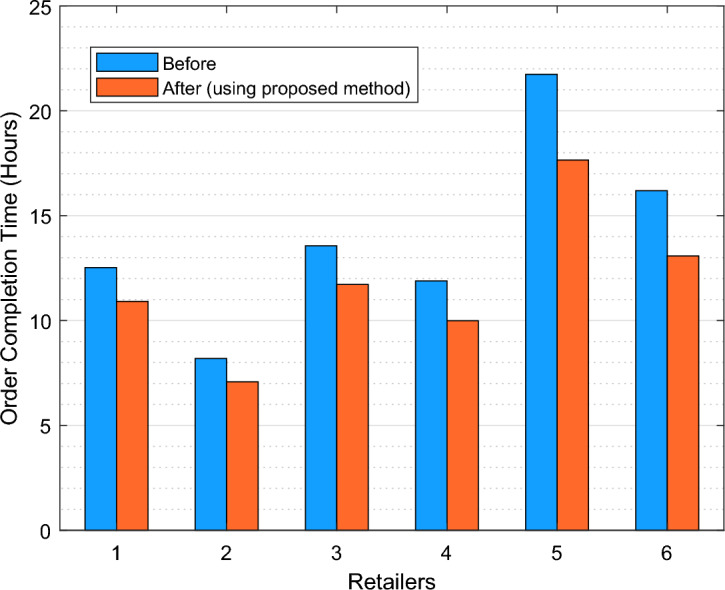
The impact of the proposed model on increasing CBEC efficiency from the perspective of order delivery time.

Figure 2 illustrates the effect that the proposed model has on enhancing the efficacy of CBEC in terms of delivery time. An analysis was performed on the mean completion time of each of the six retailers whose data was being examined. The results indicated that the execution of the proposed framework effectively reduced the duration required to complete orders for each retailer. The implementation of the suggested model did, in fact, decrease the average order completion time by 15.37%. Using a proactive approach to inventory management for retailers in the proposed model has increased the system's efficacy and enhanced the performance of the supply chain system, according to these findings.

### Second phase: performance analysis of sales prediction

The assessments conducted within this segment evaluate the performance of the proposed method in predicting sales. In the proposed procedure, the efficacy of the model was assessed using three distinct modalities. The proposed method, in which the intended prediction is derived from the outputs of ANN models, CapSA, and their weighted combination, is one of these approaches. An alternative mode is the Conventional Ensemble, which predicts the target variable using a base ensemble algorithm. Simple averaging is considered as an alternative to weighted voting and the determination of optimal weights when attempting to predict the objective variable. The third mode, known as the Single ANN (SCG), entails training a conventional ANN model with the SCG function in order to generate predictions for the target variable. Put differently, it signifies the application of a basic ANN model featuring a static topology and SCG training function in order to make predictions on the target variable.

**Figure 3:**
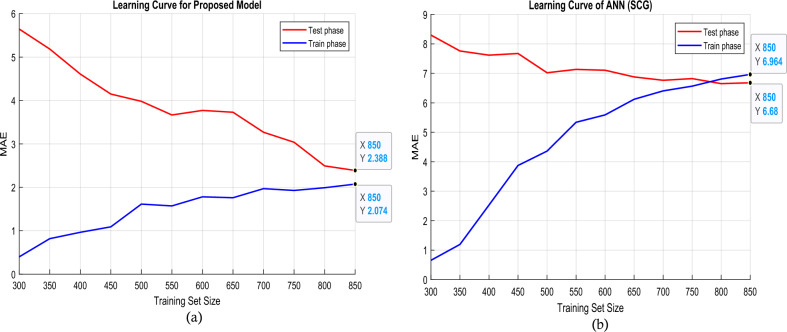
Learning curve (**a**) proposed method and (**b**) ANN with SCG training function for changes in training data size.

The efficacy of the training system is assessed in Fig. 3, which illustrates the learning curve. The learning trajectory for the proposed method during both the training and testing phases is depicted in Fig. 3a. Comparable outcomes were observed when the performance of the conventional ANN was evaluated utilizing the SCG training function, as shown in Fig. 3b. The mean absolute error is the metric that is taken into account in the learning curves. When ANN models are configured utilizing an optimization algorithm, the model's training structure approaches its ideal state. In other words, as the number of training data increases, the testing error reduces dramatically and approaches the training error. The gap between training and testing errors narrows to an acceptable level, showing that the model based on this structure is generally generalizable and can be used to a wide range of data. However, as shown in Fig. 3b, when the SCG function is employed and the number of training data rises, so does the training error, but the reduction in testing error is smaller than that of the suggested technique. This suggests that using the SCG function in constructing fixed ANN models increases the danger of overfitting. In other words, a model developed performs sufficiently during the training phase but fails to offer the desired decrease in error for testing data, resulting in a lack of generalizability. As a consequence, the suggested structure for optimizing the training of ANN models is more generalizable than standard training structures, which the learning curve structure confirms.

**Figure 4:**
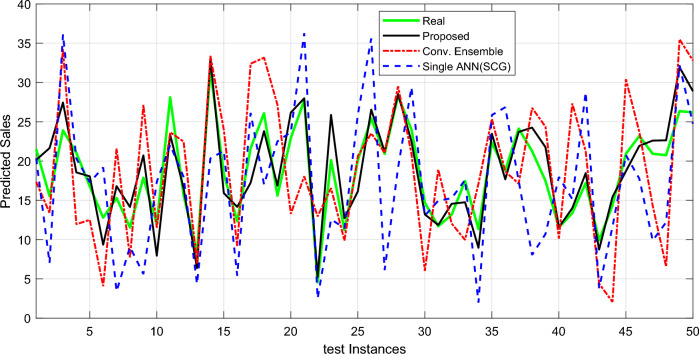
Number of registered orders versus values predicted by the proposed method and other states for 50 test samples.

Figure 4 shows an example of how the suggested technique predicts each of its modes. The green line reflects the actual performance of the proposed approach in sales forecasting, whereas the other lines indicate other modes. This graph clearly illustrates that the suggested technique gives findings that are more consistent with real-world sales performance, and the discrepancy across modes is reduced. It should be noted that these findings are based on 50 samples from the database.

**Figure 5:**
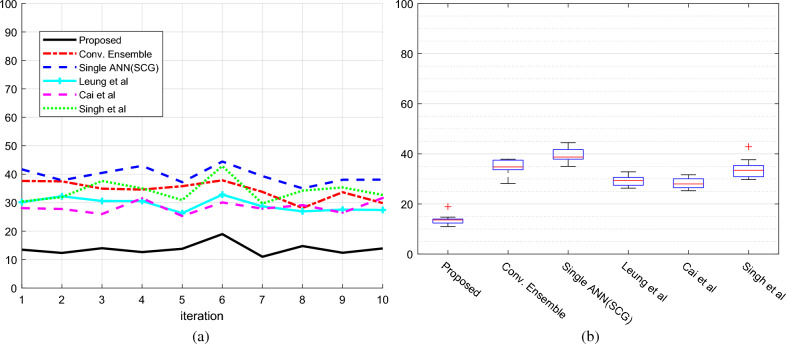
Prediction error of order volume based on MAPE (**a**) error in each cross-validation iteration (**b**) box plot of the error of each method.

Figure 5a depicts the mean absolute percentage error (MAPE) for the sales prediction assessment over iterations, both individually and aggregated. The suggested strategy achieved the lowest error value of 13.72, beating previous similar techniques. The MAPE was calculated as the mean percentage error between planned and actual values. Figure 5b depicts the error change intervals using boxplot graphs. In this graph, each method's performance is separated into four portions, with the MAPE error presented in four distinct quadrants. The top and lower lines of each quartile show the quartile’s blue end limits, while the center of the box represents the MAPE median value. This graph shows that the suggested approach has a lower MAPE value than other methods throughout several repeats, which is extremely important. The term MAPE is defined as follows:

8 $$MAPE=\frac{1}{N}{\sum }_{i=1}^{N}\frac{\left|{y}_{i}-{\widehat{y}}_{i}\right|}{{y}_{i}}$$

where ${y}_{i}$ is the product's actual sales, ${\widehat{y}}_{i}$ is the forecast value, $N$ is total number of observations.

**Figure 6:**
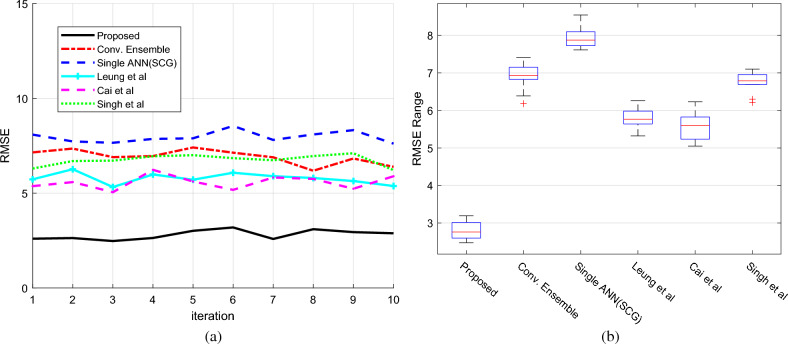
Prediction error of order volume based on RMSE (**a**) error in each cross-validation iteration (**b**) box plot of the error of each method.

Figure 6a,b displays the RMSE diagrams for several iterations. The suggested technique consistently produces lower squared error, greater accuracy, and lower RMSE than earlier conditions and methods, resulting in a higher likelihood of correct outputs. These findings indicate that the suggested strategy beats previous methods in terms of predictability and accuracy when projecting sales. Our suggested technique has a lower RMSE value than the Conventional Ensemble and Cai et al. methods, at 4.11 and 2.77, respectively. This shows that the suggested technique outperformed the other methods. The RMSE is defined as the following:

9 $$RMSE=\sqrt{\frac{1}{N}{\sum }_{i=1}^{N}{\left({y}_{i}-{\widehat{y}}_{i}\right)}^{2}}$$

where $N$ denotes the number of data samples. ${Y}_{i}$ represents the sales actual value of ith sample, and $\widehat{{\text{y}}}_{{\text{i}}}$ is the corresponding predictions of the sample using the proposed method.

**Figure 7:**
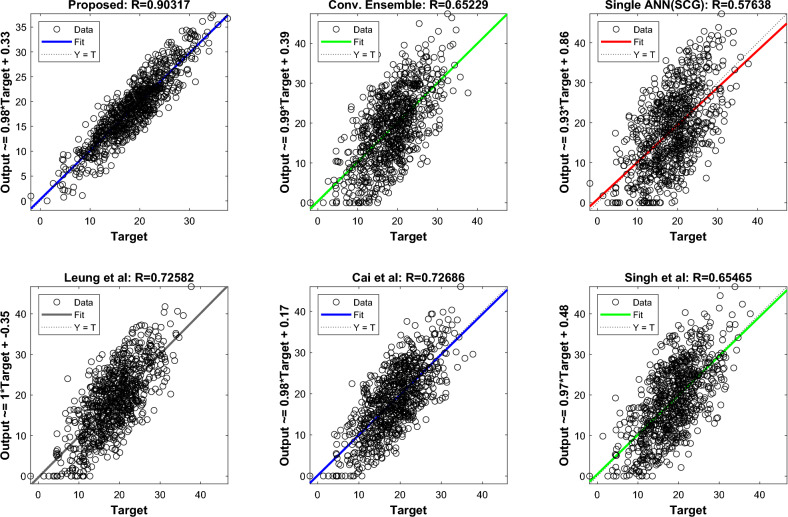
Regression charts of different methods in predicting customer order volume.

Regression diagrams depicting the correlation between predicted and actual output values are illustrated in Fig. 7. As mentioned earlier, the R value of the suggested approach is greater than that of the Conventional Ensemble and Cai et al. methods, which have R values of 0.25 and 0.18, correspondingly. This exemplifies the performance of the proposed method with respect to the R value.

**Figure 8:**
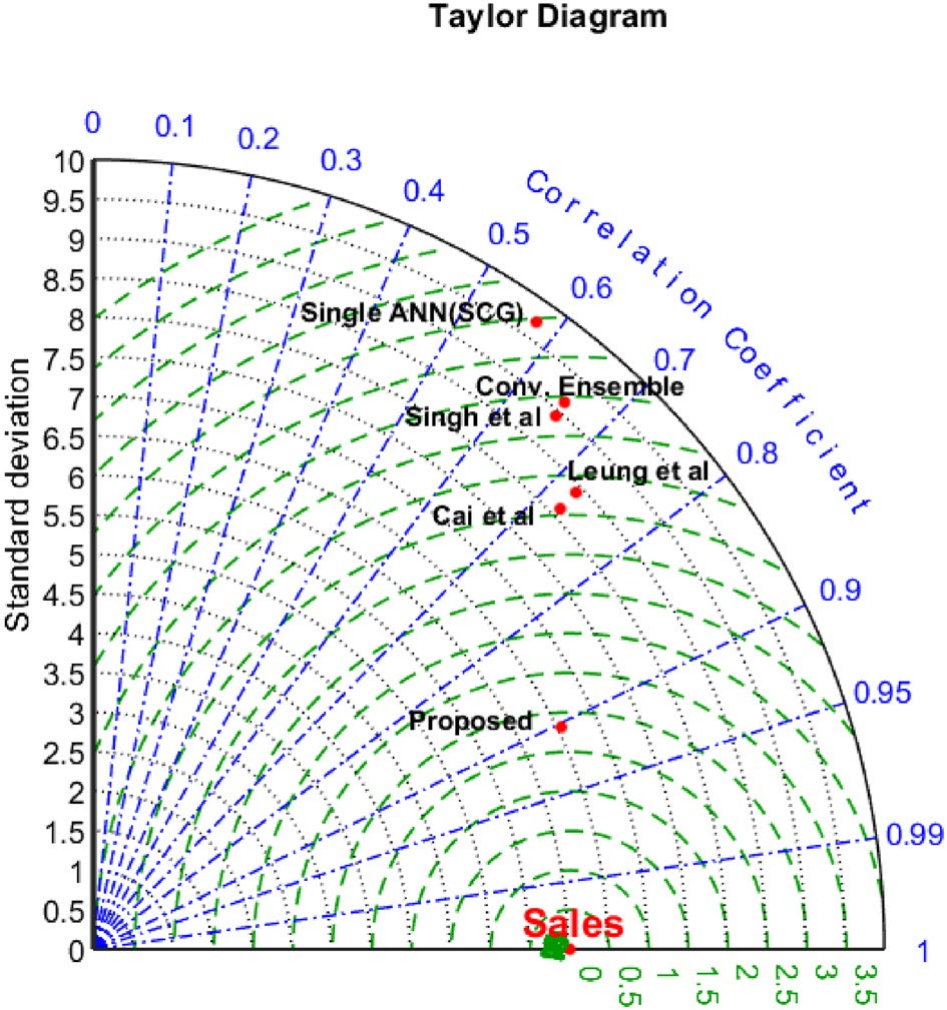
Taylor diagram resulting from comparing the performance of different methods.

A Taylor diagram is presented in Fig. 8 that illustrates the comparison of multiple approaches based on three criteria: RMSE, standard deviation, and correlation coefficient. As these graphs unequivocally demonstrate, the suggested approach boosted the correlation by a minimum of 0.18%. Furthermore, it has had a considerable impact in lowering the correlation coefficient. Furthermore, the disparity between the anticipated outputs of the proposed technique and the actual outputs (i.e., sales) has reduced, with a reduction of around 0.25, which is significant when compared to the closest method, Cai et al.

Table 2 compares several methodologies according to assessment measures. The proposed technique has the lowest MAE and RMSE, as well as a high correlation coefficient (R) of 0.9031, suggesting superior predictive accuracy. The Conventional Ensemble approach has greater MAE and RMSE values than the Single ANN method. Leung et al. and Cai et al. approaches are highly predictive, but the Singh et al. method falls short. In general, the suggested technique is the most accurate and dependable according to the assessment criteria.

**Table 2:** Summary of results obtained from experiments.

Methods	MAE	RMSE	NRMSE	MAPE	R (Correlation)
Proposed	2.2773	2.8144	0.0711	13.7211	0.9031
Conventional Ensemble	5.6761	6.9286	0.1751	34.3784	0.6522
Single ANN (SCG)	6.7171	7.9662	0.2013	39.5184	0.5763
Leung et al	4.7893	5.7856	0.1462	29.3341	0.7258
Cai et al	4.6769	5.5828	0.1411	28.3972	0.7268
Singh et al	5.5415	6.7567	0.1708	34.0769	0.6546

## Conclusion

The research offered a novel way for improving supply chain efficiency in the CBEC business by using machine learning techniques and the Internet of Things. The recommended technique was designed to accurately estimate client demands and maximize product distribution, resulting in cost savings. The order prediction and allocation operation phases were the two key components of the technique. Using a combination of the CapSA and weighted neural networks, the technique showed a noteworthy 2.4 decrease in RMSE when compared to comparable methods. Furthermore, compared to other comparison approaches, there was a significant decrease in MAPE of 14.67, indicating the better performance of the suggested strategy in improving supply chain operations in the CBEC sector. Consequently, the research developed a comprehensive framework for using machine learning and Internet of Things technologies to enhance supply chain performance in the CBEC area. According to the findings, the suggested strategy was very successful in forecasting sales and allocating resources, which made it a priceless resource for CBEC companies.

The article's limitations mostly concern the computational costs of the model, which arise from combining numerous models using the CapSA method. When ANN models are trained and fused using this framework, it might be more expensive and need more processing power than when using traditional training techniques like SCG. Even though there could be performance gains, this method might increase computational overheads. In addition, the complexity of integrating heterogeneous data from several IoT devices is another limitation imposed by the suggested approach. The incorporation of diverse data might increase the intricacy of the model and need the use of specialist infrastructure to ensure efficient data handling. Our future work will focus on streamlining this organization to increase productivity.

## Data Availability

All data generated or analysed during this study are included in this published article.
